# Effect on Soil Properties of* BcWRKY1* Transgenic Maize with Enhanced Salinity Tolerance

**DOI:** 10.1155/2016/6019046

**Published:** 2016-11-20

**Authors:** Xing Zeng, Yu Zhou, Zhongjia Zhu, Hongyue Zu, Shumin Wang, Hong Di, Zhenhua Wang

**Affiliations:** College of Agronomy, Northeast Agricultural University, Mucai Street, Xiangfang District, Harbin, Heilongjiang 150030, China

## Abstract

Maize (*Zea mays* L.) is the most important cereal crop in the world. However, soil salinity has become a major problem affecting plant productivity due to arable field degradation. Thus, transgenic maize transformed with a salinity tolerance gene has been developed to further evaluate its salt tolerance and effects on agronomic traits. It is necessary to analyze the potential environmental risk of transgenic maize before further commercialization. Enzyme activities, physicochemical properties, and microbial populations were evaluated in saline and nonsaline rhizosphere soils from a transgenic maize line (WL-73) overexpressing* BcWRKY1* and from wild-type (WT) maize LH1037. Measurements were taken at four growth stages (V3, V9, R1, and R6) and repeated in three consecutive years (2012–2014). There was no change in the rhizosphere soils of either WL-73 or WT plants in the four soil enzyme activities, seven soil physicochemical properties, and the populations of three soil organisms. The results of this study suggested that salinity tolerant transgenic maize had no adverse impact on soil properties in soil rhizosphere during three consecutive years at two different locations and provided a theoretical basis for environmental impact monitoring of salinity tolerant transgenic maize.

## 1. Introduction

In China, maize (*Zea mays* L.) is the most important cereal crop, and the production of this crop is affected by soil salinity. This problem has become ubiquitous in many countries. Thus, many salinity-tolerant crops, such as maize and rice, have been developed using transgenic technology [1]. Researchers have found that the microbial communities in the rhizosphere are influenced by the plant. Questions have been raised about whether antibiotic resistance genes, as selective markers, can transfer from genetically modified GM plants to indigenous microbes in the soil rhizospheres. Another question is whether certain GM plants differentially affect soil microbial communities compared to non-GM plants [2, 3].

Previous studies have shown that GM plants, including GM maize, potato, soybean, rice, and triticale, are equivalent to non-GM crops in terms of nutrition and are safe as food or feed [4]. The effects of* Bacillus thuringiensis* (*Bt*) transgenic cotton (“Mech 162”) and non-*Bt* plants of the same cultivar on the ecology of many organisms in the soil were evaluated over three years in a subtropical environment. The authors concluded that the* Bt* cotton “Mech 162” did not have any negative effects on the organisms or biochemical characteristics of the soil [5]. The bacterial communities in the rhizosphere were studied using GM and non-GM maize in another study. Plant growth can promote rhizobacterial multiplication associated with both GM and non-GM plants, which indicates the mutually beneficial relationship between rhizobacteria and maize. No significant differences in the isolated rhizospheres were found during plant growth in GM or non-GM plants [6]. Using transgenic, salinity-tolerant* SUV3* and* PDH45* rice, the communication between rhizobacteria and rice was studied, and no significant effect was found [3, 7]. However, there have been few reports of the influence of GM and non-GM maize on rhizosphere soils [8]. The* BcWRKY1* gene was cloned from* Boea crassifolia* Hemsl, and it encodes a 444-amino acid WRKY-like protein containing two conserved domains: WRKYGQK and C2H2 motifs. The full-length* BcWRKY1* cDNA was 1,803 bp, and its expression could be induced by abiotic stresses, including soil salinity, low temperature, and drought. In addition,* BcWRKY1* transcription was accompanied by changes in plant hormones, including abscisic acid (ABA), salicylic acid (SA), and jasmonic acid (JA) [9]. Then, the* Boea crassifolia* DNA helicase of* BcWRKY1* was overexpressed under salt stress in maize. The NaCl stress-tolerant phenotype appeared even when plants were irrigated continuously with 150–200 mM NaCl, with no effect on their yield. Furthermore, other salt-induced genes, including* GmWRKY54*,* TaWRKY2*, and* HvWRKY38*, in* Arabidopsis thaliana* also promoted strong NaCl stress-tolerant phenotypes [10–12].

In this study, GM maize (WL-73) plants overexpressing the* BcWRKY1* gene and control non-GM maize LH1037 were used to evaluate the effects of WL-73 growth on the microbial populations in saline or nonsaline soils in Harbin, China.* BcWRKY1*-transgenic maize, which carries a kanamycin resistance gene, was compared to non-GM LH1037 maize to determine its effects on rhizosphere soil in terms of enzyme activities (including dehydrogenase, alkaline phosphatase, urease, and sucrase activities), physicochemical properties, and microbial populations.

## 2. Materials and Methods

### 2.1. Plant Sample Treatment

**Figure 1:**
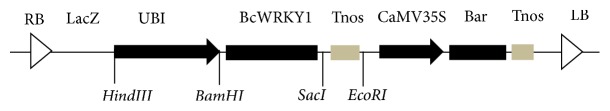
Schematic of the expression vector p3300-Ubi-*BcWRKY1*. RB, right border; LB, left border; UBI, ubiquitin promoter; Tnos, nopaline synthase terminator;* BcWRKY1*,* Boea crassifolia WRKY1* gene; Bar, Bialaphos resistance selectable marker gene;* CaMV35S*, cauliflower mosaic virus 35S promoter.* HindIII*,* BamHI*,* SacI*, and* EcoRI* are restriction endonuclease recognition sites.

The line WL-73 was derived from the maize inbred line LH1037 transformed with the vector pCAMBIA 3300-*Ubi-WRKY1* (Figure 1). Seeds were obtained from T0 antisaline transgenic maize selected by saline stress and self-crossed to T6; WL-73 plants overexpressing* BcWRKY1* were able to survive under 300 mM NaCl stress [13].

The seeds of WL-73 and its receptor line LH1037 (WT) were grown in a three-row field with simulated saline-alkaline soil derived from a natural saline-alkaline field in Heilongjiang Province, China. The saline soil contained 29.42 g·kg^−1^ organic matter, 0.31 g·kg^−1^ total N, 127.27 mg·kg^−1^ available N (AN), 23.54 mg·kg^−1^ available P (AP), and 178.91 mg·kg^−1^ available K (K_2_O) (AK) and had a pH of 8.65. The nonsaline control soil contained 58.02 g·kg^−1^ organic matter, 0.31 g·kg^−1^ total N, 119.26 mg·kg^−1^ AN, 26.01 mg·kg^−1^ AP, and 267.14 mg·kg^−1^ AK and had a pH of 7.67.

The seeds were salt and mock treated following Di's method with modifications [14]. WT and WL-73 seeds were germinated in sterilized vermiculite in a greenhouse with a humidity of 40–50% at 22°C and a light cycle of 16 h light/8 h darkness. The plants were well watered until the three-leaf stage. In addition, 0.5x Hoagland's nutrient solution with 300 mmol NaCl was applied to the salt treatment plants daily for 7 days, while the same solution without NaCl was applied to the control plants at the same frequency. Both the salt-treated and control plants in the experiment were then watered with 0.5x Hoagland's nutrient solution every 3 days to prevent excessive NaCl accumulation in the vermiculite.

### 2.2. Molecular Characterization and Salt Tolerance of Transgenic Maize

Leaves of the salt-treated seedlings were collected, and DNA and RNA were isolated. The CTAB method was used to isolate genomic DNA from the two youngest leaves of each plant [15]. Total RNA was isolated using TRIzol following the manufacturer's protocol (Tiangen Biotech, Beijing, China) under the requirement of 100 mg of young seeding leaves per mL of TRIzol. Exogenous* BcWRKY1* gene transcription was analyzed by RT-PCR.

The plant height and fresh weights were measured according to the methods of Di [14]. In addition, the membrane integrity parameters of the plants were determined by detecting superoxide dismutase (SOD) and peroxidase (POD) activity, proline (Pro) and malondialdehyde (MDA) content, relative electrical conductivity (REC), and chlorophyll content in leaves following the methods of Arnon [16] and Bates et al. [17].

### 2.3. Rhizosphere Soil Sampling

WL-73 and WT maize plants were grown in saline or conventional soil in triplicate from 2012 to 2014 at the Transgenic Experiment Station of Northeast Agricultural University, Harbin, Heilongjiang, China (longitude 126°73′, latitude 45°75′). Soil samples were isolated from the rhizosphere of WL-73 and WT at the V3 (the three lowest leaves have a visible collar), V9 (nine leaves have collars present), R1 (silking), and R6 (physiological maturity) stages. After removing the surface leaves, three soil samples from each plot were collected according to a checkerboard method. The soil volumes between 0 and 20 cm in depth were extracted using a soil auger with a 4 cm diameter, and the bulk soil on the root was shaken off. The soil from the root was stripped using a sterilizing brush and constituted the rhizosphere soil samples. We mixed three rhizosphere soil samples from each plot into one sample and then divided this sample into two. One of the samples was stored at 4°C until microbial analysis. Another sample was air-dried at room temperature, homogenized by sieving through a 2 mm mesh, and stored at 4°C until analysis.

### 2.4. Measurement of Soil Enzyme Activities

Dehydrogenase activity was analyzed as described by Min et al. [18]. Alkaline phosphatase activity was measured spectrophotometrically as described by Tabatabai and Bremner [19]. Urease enzyme activity was estimated as previously described [20]. Soil sucrase activity was measured using the 3,5-dinitrosalicylic acid method [21, 22].

### 2.5. Quantification of Physicochemical Properties

The physicochemical characteristics of soil and nutrient constituents, including soil type, pH, electrical conductivity (Ec) (mS·cm^−1^), organic carbon (OC) (%), AN (kg·ha^−1^), AP (kg·ha^−1^), and AK (kg·ha^−1^), were determined.

The soil pH and Ec were analyzed through the following steps. A 50 g soil sample was suspended in 100 mL of distilled, deionized water and stirred for 1 h at 100 rpm on a rotary shaker. The supernatant was collected by centrifugation at 10,000 ×g for 5 min. The Ec was recorded using a conductivity meter against 0.01 N KCl, and the pH was measured [23].

The available carbon, nitrogen, phosphorus, and potassium contents in the soils were determined following standard methods [24–27].

The calcium (Ca^2+^), sodium (Na^+^), and magnesium (Mg^2+^) ion concentrations were determined using an atomic absorption method to determine the sodium adsorption ratio (SAR) [26]. The SAR was then calculated using the following formula:

(1) $$\begin{array}{c} SAR=\frac{{Na}^{+}}{\sqrt{\left(1/2\right)\left({Ca}^{2+}+{Mg}^{2+}\right)}}. \end{array}$$

### 2.6. Isolation of Rhizospheric Bacteria, Actinomycetes, and Fungi

To obtain isolated colonies, serial dilutions (10^−4^ dilution) prepared from 1 g soil samples were streaked onto nutrient agar medium in plates. Colonies were then selected, diluted, and spread onto plates containing beef extract peptone agar to detect bacteria, Gause's agar to detect actinomycetes, and Rose Bengal agar to detect fungi. Three replicates of the inoculated agar plates were incubated at 30°C, 28°C, or 28°C for 3 d for bacteria, 3 d for actinomycetes, and 5 d for fungi, after which the number of various types of colonies was recorded. The total populations of bacteria, actinomycetes, and fungi in each Petri dish were counted as colony forming units (cfu)·g^−1^ dry soil.

### 2.7. Data Processing Methods

This study was designed as a randomized complete block. The block treatments were the four growth stages (V3, V9, R1, and R6), the two maize materials (WL-73 and WT), and the two soil types (saline and nonsaline). All of the experiments were performed with three biological replicates over three years from 2012 to 2014. The data were analyzed statistically, and the standard error was calculated. An analysis of variance (ANOVA) was performed on treatment means using a generalized linear mixed model (GLMM), including treatment and sample time, in SAS 9.1 (Copyright 2008, SAS Institute, Cary, NC). Mean separations were performed using a least significant difference (LSD) test.

## 3. Results and Discussion

### 3.1. Molecular Characterization of Transgenic Maize Plants (WL-73)

The* BcWRKY1* gene was cloned from* Boea crassifolia*, which has the ability to tolerate salt stress [9]. WL-73 transgenic plants overexpressing the* BcWRKY1* gene were successfully generated by* Agrobacterium*-mediated transformation with the binary vector pCAMBIA3300-Ubi-*BcWRKY1* (Figure 1) introduced into the inbred line LH1037 [28].

**Figure 2:**
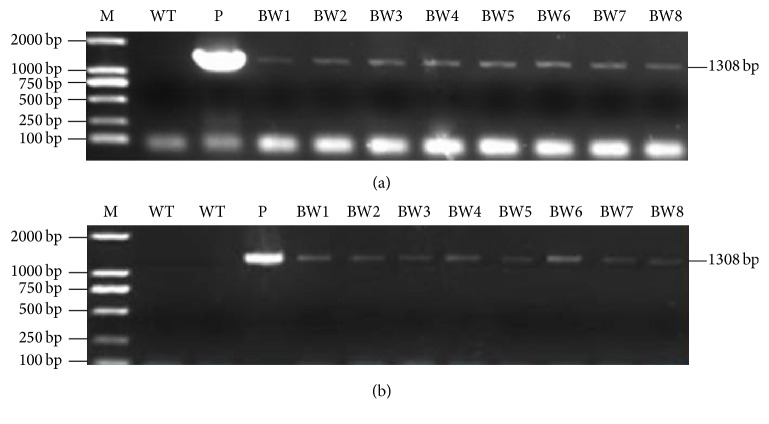
PCR and RT-PCR analyses of transformed and WT plants using* BcWRKY1* primers. (a) PCR analysis of WL-73 and WT; M, DNA marker DL2000 (TaKaRa); WT, wild-type plants of LH1037; P, pCAMBIA3300-*Ubi-WRKY1* plasmid; BW1-8 derived from plants of WL-73. (b) RT-PCR analysis of WL-73 and WT1; M, DNA marker DL2000 (TaKaRa); WT, wild-type plants of LH1037; P, pCAMBIA3300-*Ubi-WRKY1* plasmid; BW1-8 derived from WL-73 plants.

The 1308-bp* BcWRKY1* PCR product was amplified from WL-73 transgenic plants with* BcWRKY1* gene-specific primers (Figure 2(a)). The transcription of the* BcWRKY1* gene in plant leaves was detected by RT-PCR (Table 1, Figure 2(b)). As expected, PCR and RT-PCR bands characteristic of* BcWRKY1* were detected in WL-73 but not in WT plants.

### 3.2. Salt Tolerance Evaluation of Transgenic Maize Plants (WL-73)

**Figure 3:**
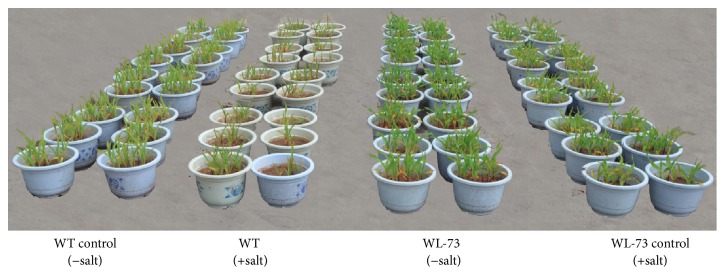
Enhanced salt tolerance of transgenic versus wild-type seedlings under 300 mM NaCl. Wild-type and transgenic maize plants were treated with 0.5x Hoagland's nutrient solution and either 0 mM NaCl (control) or 300 mM NaCl for 7 days. WL-73, LH1037 plants transformed with* BcWRKY1*; WT, wild-type LH1037 plants. Note: salt tolerance of transgenic maize compared to WT. Photographs were taken after salt treatment.* BcWRKY1*-overexpressing T6 transgenic and WT maize plants under salt-stressed (300 mM NaCl) and nonstressed conditions after 7 days.

**Figure 4:**
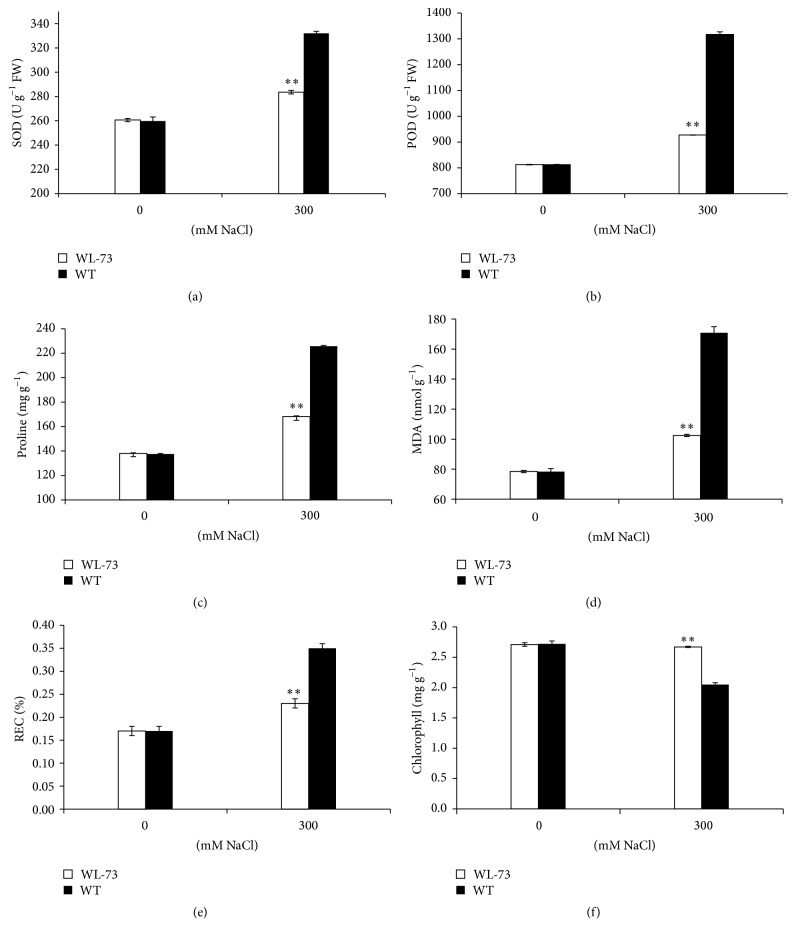
Effects of NaCl stress on the SOD, POD, Pro, MDA, REC, and chlorophyll contents of WT maize and WL-73 transgenic maize plants. WT and WL-73 were treated with 0.5x Hoagland's nutrient solution and either 0 mM NaCl (control) or 300 mM NaCl for 7 days; then, the SOD, POD, Pro, MDA, REC, and chlorophyll contents were measured. (a) SOD content; (b) POD content; (c) Pro content; (d) MDA content; (e) REC; (f) chlorophyll content. WT, wild-type maize LH1037; WL-73, LH1037 plant transformed with* BcWRKY1. ∗∗* indicates a significant difference at 0.01 according to the LSD test (*n* = 3). The standard error is based on the average of three biological replicates. Note: the SOD, POD, Pro, MDA, and REC of WL-73 were significantly lower than those of WT (*P* < 0.01), while the chlorophyll content of WL-73 was higher than that of WT under 300 mM NaCl treatment for 7 days.

When we treated maize plants with 300 mM NaCl solution for 7 days, the WL-73 plants were 5.3 cm taller and 60% heavier (fresh weight) than WT plants (Table 2). WT seedlings became almost entirely yellow on the 7th day after salt stress (Figure 3). The membrane integrity of the plants was measured in terms of parameters such as SOD, POD, Pro, MDA content, REC, and chlorophyll content following salt stress (Figure 4). The SOD, POD, Pro, MDA, and REC of WL-73 plants were significantly lower than those of WT plants (*P* < 0.01), while the chlorophyll content of WL-73 plants was higher than that of WT plants under the 300 mM NaCl treatment (Table 2). However, no significant difference was found in these values between WL-73 and WT plants under control conditions. These results suggest that the membranes of WL-73 plants were less damaged than those of the WT plant.

Our results suggest that* BcWRKY1* enhanced the tolerance of WL-73 plants to salinity stress via membrane stabilization and reduced REC and MDA contents compared with WT plants under salt stress (Figure 4, Table 2).

The* BcWRKY1* gene has been analyzed with other stress-related genes in transgenic plants, where it enhances the tolerance to salt and drought stress [10–12]. Therefore, increasing the expression level of* BcWRKY1* via transgenic technology should be critical for engineering crop plants with improved tolerance under multiple environmental stresses.

### 3.3. Activities of Four Enzymes in Rhizosphere Soil

**Figure 5:**
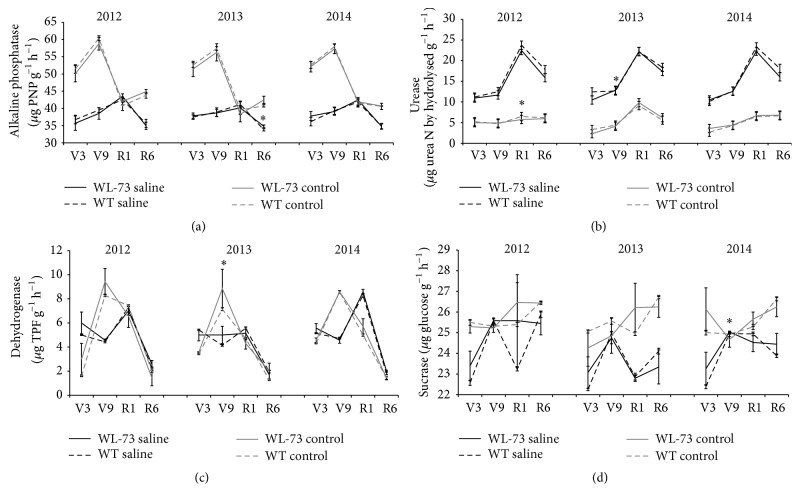
Activities of four enzymes in rhizosphere soil at different sampling times. (a) Alkaline phosphatase activity; (b) urease activity; (c) dehydrogenase activity; (d) sucrase activity. WT, wild-type maize LH1037; WL-73, LH1037 plant transformed with* BcWRKY1*; V3, the three lowest leaves have a visible collar; V9, nine leaves have collars present; R1, silking; R6, physiological maturity. *∗* indicates a significant difference at *P* < 0.05 according to the LSD test (*n* = 3). The standard error is based on the average of three biological replicates. Note: during the three years of this study, there were no overall significant differences (*P* > 0.05) in the alkaline phosphatase, urease, dehydrogenase, or sucrase activity in rhizosphere soil of WL-73 and WT plants in two soil environments (saline or nonsaline) or at four growth stages (V3, V9, R1, and R6). In addition, the activities of these four soil enzymes were not significantly different between WL-73 and WT plants in some soil environments, years, and growth stages.

The effects of WL-73 maize compared to control maize on rhizosphere soil enzyme activity, including the activities of alkaline phosphatase, urease, dehydrogenase, and sucrase, were studied in saline or control soil environments at four maize growth stages (V3, V9, R1, and R6) from 2012 to 2014. These four enzymes are the main enzymes in soil and significantly affect the growth and yield of maize. Alkaline phosphatase is mainly involved in the soil phosphorus cycle. Urease is associated with the nitrogen cycle. Dehydrogenase is the main oxidoreductase, and sucrose is the major hydrolase enzyme. During the three years of this study, the alkaline phosphatase, urease, dehydrogenase, and sucrase activities in the rhizosphere soil of WL-73 and WT plants were not different (*P* > 0.05) in the two soil environments (saline or nonsaline) or at any of the four growth stages (V3, V9, R1, and R6). The ANOVA results showed that the dehydrogenase and alkaline phosphatase activities in the rhizosphere soil of WL-73 and WT maize were similar, with values ranging from 34.84 to 39.04 *μ*g PNP g^−1^·h^−1^ in saline soil and from 40.74 to 57.44 *μ*g PNP g^−1^·h^−1^ in control soil for WL-73 and from 34.74 to 41.85 *μ*g PNP g^−1^·h^−1^ in saline soil and from 40.62 to 59.26 *μ*g PNP g^−1^·h^−1^ in control soil for WT maize (Tables 3 and 4; Figures 5(a)–5(d)). However, the activities of these four soil enzymes were significantly different between WL-73 and WT plants in some soil environments, years, and growth stages. For example, there were significant differences in alkaline phosphatase activity in saline soil at R6 in 2013 (*P* = 0.03); in urease activity in control soil at R1 in 2012 and in saline soil at V9 in 2013 (*P* < 0.05); in dehydrogenase activity in control soil at V9 in 2013; and in sucrase activity in saline soil at V9 in 2014 (Table 3, Figures 5(a)–5(d)). However, no consistent trends in enzyme activity were detected in the two soil environments, over the three-year study, or in the four growth stages analyzed here. These results agree with those of previous studies in other regions and for a variety of crops [29–32].

Soil enzymes play an important role in maintaining soil ecology, physicochemical properties, fertility, and health [33, 34]. The overexpression of* PDH45* and* SUV3* in transgenic rice has no adverse effect on rhizosphere soil or its microflora [3, 7]. In other studies of transgenic crops, the only consistent significant differences in soil enzymes and physicochemical properties between transgenic and nontransgenic plants were due to seasons and crop varieties. There were no significant differences in the enzyme activities of rhizospheric microbes from soils in which* Bt* or non-*Bt* cotton was grown [5]. The results of the present study indicated few significant differences in the alkaline phosphatase, urease, dehydrogenase, and sucrase activities in the rhizosphere soil between WL-73 and WT plants, as in the five studies cited above.

### 3.4. Physicochemical Properties of Rhizosphere Soil

**Figure 6:**
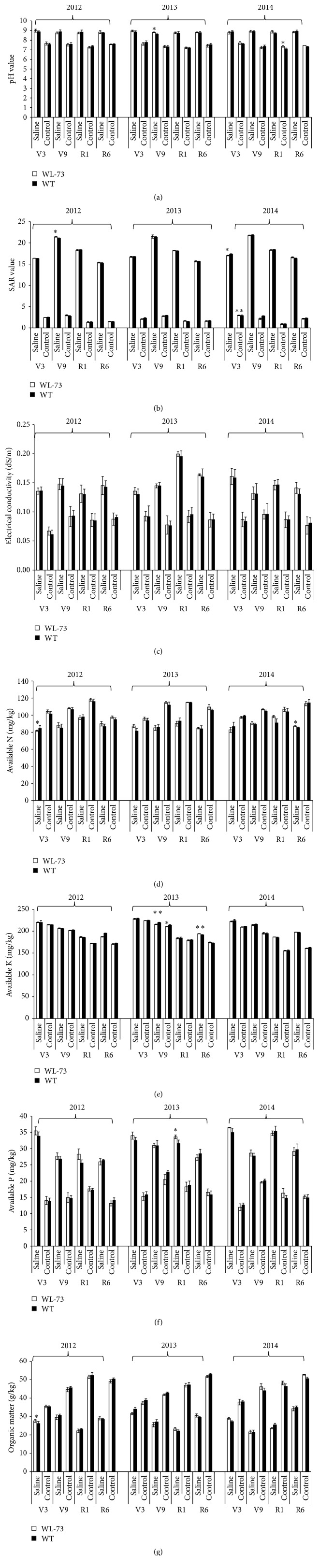
Assays of seven physicochemical properties of rhizosphere soil.* BcWRKY1* transgenic maize (WL-73) and nontransgenic maize (WT; LH1037) plants were grown in saline or control nonsaline soil from 2012 to 2014. Seven physicochemical parameters were measured, and a statistical analysis was performed using the LSD test with *P* < 0.05 and *n* = 3. (a) pH; (b) SAR value; (c) electrical conductivity; (d) available N; (e) available K; (f) available P; (g) organic matter. WT, wild-type maize LH1037; WL-73, LH1037 plant transformed with* BcWRKY1*; V3, the three lowest leaves have a visible collar; V9, nine leaves have collars present; R1, silking; R6, physiological maturity. *∗* indicates a significant difference at *P* < 0.05 according to the LSD test (*n* = 3). *∗∗* indicates a significant difference at *P* < 0.01 according to the LSD test (*n* = 3). The standard error is based on the average of three biological replicates. Note: no overall significant differences were observed between WL-73 and WT plants for most of the physicochemical properties (*P* > 0.05) at four maize growth periods (R1, R9, V1, and V6) in saline and control soil environments from 2012 to 2014.

The physicochemical properties, including the pH, SAR, Ec, AN, AK, AP, and OC, of rhizosphere soil from WL-73 and WT maize plants are shown in Figures 6(a)–6(g). There were no overall significant differences between WL-73 and WT plants for most of the physicochemical properties (*P* > 0.05) at four maize growth periods (R1, R9, V1, and V6) in saline and control soil environments from 2012 to 2014 (Table 5). However, there were significant differences between WL-73 and WT plants (*P* < 0.05) for nine combinations of factors and very significant differences between the genotypes for three combinations of factors. There were significant differences in specific parameters under some conditions, including pH in saline soil at V9 in 2013; pH in control soil at R1 in 2014; SAR in saline soil at V9 in 2012 and 2014; AN in saline soil at V9 in 2013 and in saline soil at R6 in 2014; AK in control soil at V9 in 2013; AP in saline soil at R1 in 2013; and organic matter in saline soil at V9 in 2013. There were very significant differences in SAR in control soil at V9 in 2014; AK in saline soil at V9 in 2013; and AK in saline soil at R6 in 2013 (Table 3; Figures 6(a)–6(g)). However, no overall consistent trends in physicochemical properties were detected in the two soil environments, over the three-year study, or in the four growth stages. This result is consistent with previous results in other plants and fields [32].

### 3.5. Culturable Microbial Populations in Rhizosphere Soil

**Figure 7:**
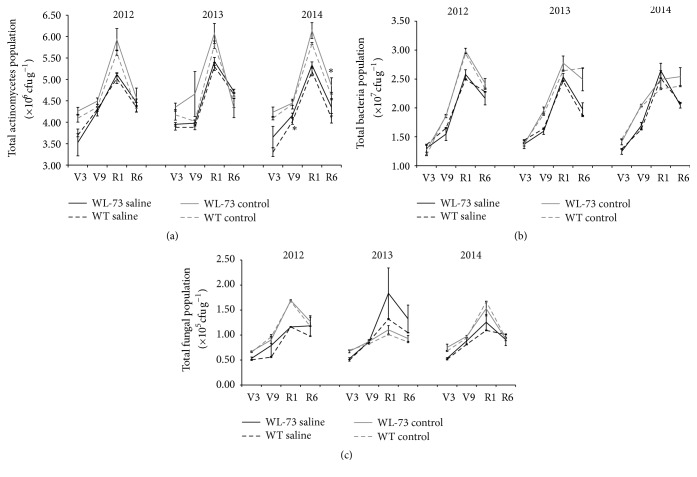
Assays of three microbial communities in rhizosphere soil.* BcWRKY1* transgenic maize (WL-73) and nontransgenic maize (WT; LH1037) plants were grown in saline or control nonsaline soil from 2012 to 2014 (^*∗*^ *P* < 0.05, *n* = 3). We investigated the microbial populations in their rhizosphere soil. (a) Actinomycete population, (b) bacterial population, and (c) fungal population in terms of cfu (colony forming units) per g of dry soil. WT, wild-type maize LH1037; WL-73, LH1037 plant transformed with* BcWRKY1*; V3, the three lowest leaves have a visible collar; V9, nine leaves have collars present; R1, silking; R6, physiological maturity. *∗* indicates a significant difference at *P* < 0.05 according to the LSD test (*n* = 3). The standard error is based on the average of three biological replicates from 2012 to 2014. Note  1: the variation in the actinomycete, bacterial, and fungal populations was consistent. The populations of the three microbial communities increased from growth stages V3 through V9. The total number of each of the three types of microbes in the rhizosphere soil peaked at the V1 stage. Subsequently, the population levels of all three types of microorganisms decreased at the V6 stage. Note  2: to ensure consistency in the experimental data, transgenic and nontransgenic material were planted in the same pot for the three years, and each pot was handled at the same time to minimize the impact of human factors on the experiment.

The number of total actinomycetes, bacteria, and fungi per gram of dry rhizosphere soil from WL-73 and WT plants over the crop developmental cycle is shown in Figure 7. No significant differences were found in the total number of bacteria, fungi, and actinomycetes between the two maize rhizosphere soils at any of the plant growth stages, except for actinomycetes in saline soil at V9 and in control soil at R6 in 2014 (Table 3). The variation in actinomycete, bacterial, and fungal populations was consistent. The three microbial populations increased from growth stages V3 through V9. The total number of each of the three kinds of microbes in the rhizosphere soil peaked at the V1 stage. Subsequently, the populations of all three types of microorganisms decreased at the V6 stage (Figures 7(a)–7(c)).

Soil microbial analysis is a common method used to detect the effect of exogenous chemicals or environmental pollutants on soil fertility and crop yields. Similarly, monitoring soil microbial populations in response to transgenic plants will reveal the risks of exogenous genes in soil. Investigations of the microbial populations of rhizosphere soil found that* Bt* maize had no direct effect on soil ecology [34]. Both the number and the diversity of microorganisms exhibit only significant seasonal variation, with no long-term effect on the cultivation of Cry1Ac-transgenic cotton [32]. No significant effects were found on the populations of various soil microorganisms with the growth of transgenic insect-resistant maize,* Bt* maize, and cotton compared to nontransgenic plants under field conditions [30, 31, 35–37]. There was no adverse effect on soil enzymatic activities or rhizosphere microbial communities by the cultivation of transgenic plants, such as* MCM6* transgenic tobacco,* PDH45* transgenic rice, and* SUV3*-overexpressing transgenic rice [3, 7, 37].

In our study, significant variation was detected in actinomycete populations in saline soil at V9 and in control soil at R6 in 2014 (*P* > 0.05). However, we did not find a significant effect on enzyme activities, physicochemical properties, or populations of soil microbes due to the long-term cultivation of WL-73 compared to WT. Our results are consistent with previous studies showing that the long-term cultivation of salt-tolerant GM plants has no effect on soil microbial populations. The effects that we observed were due to particular individual plants, techniques, exogenously expressed proteins, or environmental conditions.

## 4. Conclusions

In the present study, the minor significant differences in the rhizosphere soil between transgenic and nontransgenic maize plants were not as large as the effects associated with plant growth stages. These results indicated that the effects of* BcWRKY1* maize WL-73 on rhizosphere soil ecology are within the variation expected in conventional agriculture. The long-term planting (3 years) of WL-73 plants had no detectable effects on the enzymatic activities, physicochemical properties, or microbial populations of the rhizosphere soil compared with the WT at any of four maize growth stages (V3, V9, R1, and R6).

## Figures and Tables

**Table 1:** PCR and RT-PCR primers for amplifying *BcWRKY1*.

Primer name	Sequence (5′-3′)
Primer I	ATGTCGTCTCTCGGCTCATC
Primer II	GAGCCCAACTGATTTTCTTG

**Table 2:** Height and fresh weight of maize WL-73 and WT under 300 mM NaCl stress.

Parameter	7 days of non-NaCl stress		7 days of NaCl stress	
	WL-73	WT	WL-73	WT
Height (cm)	25.7 ± 0.42^a^	25.6 ± 0.34^a^	24.4 ± 0.70^a^	19.1 ± 0.81^b^
Fresh weight per plant (g)	5.95 ± 0.21^a^	5.89 ± 0.14^a^	5.41 ± 0.63^a^	3.38 ± 0.22^b^
SOD (U·g^−1^ FW)	260.60 ± 1.35^a^	259.80 ± 1.48^a^	283.59 ± 3.38^a^	332.02 ± 1.73^b^
POD (U·g^−1^ FW)	813.03 ± 0.19^a^	812.48 ± 0.47^a^	927.62 ± 1.40^a^	1,316.92 ± 1.83^b^
Pro (mg·g^−1^)	138.00 ± 0.48^a^	137.58 ± 0.57^a^	168.17 ± 2.64^a^	225.80 ± 3.14^b^
MDA (nmol·g^−1^)	78.41 ± 0.72^a^	78.33 ± 0.75^a^	102.44 ± 2.11^a^	170.89 ± 4.06^b^
REC (%)	0.17 ± 0.01^a^	0.17 ± 0.01^a^	0.23 ± 0.01^a^	0.35 ± 0.02^b^
Chlorophyll content (mg·g^−1^)	2.71 ± 0.03^a^	2.72 ± 0.01^a^	2.67 ± 0.05^a^	2.05 ± 0.03^b^

Wild-type or transgenic maize plants were treated with 0.5x Hoagland's nutrient solution and 0 mM NaCl (control) or 300 mM NaCl for 7 days. Then, the height, fresh weight, and SOD, POD, Pro, MDA, REC, and chlorophyll contents were measured. WT, wild-type maize LH1037; WL-73, LH1037 plant transformed with *BcWRKY1*.

Different letters following the numbers in the same column indicate a significant (*P* ≤ 0.05) difference between treatments according to the LSD test.

**Table 3:** ANOVA of the effects of year, growth stage, soil type, and maize genotype.

Number	Source	Year	Growth stage	Soil type	WL-73	WT	*P *value
1	pH	2013	V9	Saline	8.83	8.66	0.03^*∗*^
2	pH	2014	R1	Control	7.36	7.13	0.02^*∗*^
3	SAR	2012	V9	Saline	21.43	21.07	0.01^*∗*^
4	SAR	2014	V9	Saline	17.02	17.33	0.03^*∗*^
5	SAR	2014	V9	Control	2.14	2.78	0.01^*∗∗*^
6	AN	2013	V9	Saline	87.33	81.69	0.05^*∗*^
7	AN	2014	R6	Saline	87.33	85.60	0.03^*∗*^
8	AK	2013	V9	Saline	215.83	219.58	0.01^*∗∗*^
9	AK	2013	V9	Control	210.45	213.99	0.02^*∗*^
10	AK	2013	R6	Saline	194.23	191.55	0.01^*∗∗*^
11	AP	2013	R1	Saline	33.63	31.66	0.04^*∗*^
12	OC	2013	V9	Saline	31.59	33.96	0.03^*∗*^
13	Alkaline phosphatase	2013	R6	Saline	34.79	33.80	0.03^*∗*^
14	Urease activity	2012	R1	Control	5.76	6.49	0.04^*∗*^
15	Urease activity	2013	V9	Saline	12.87	12.65	0.05^*∗*^
16	Dehydrogenase activity	2013	V9	Control	8.86	7.14	0.03^*∗*^
17	Sucrase activity	2014	V9	Saline	25.04	24.96	0.02^*∗*^
18	Actinomycetes	2014	V9	Saline	4.16	4.02	0.05^*∗*^
19	Actinomycetes	2014	R6	Control	4.85	4.61	0.05^*∗*^

SAR, sodium adsorption ratio; AN, available nitrogen; AK, available potassium; OC, organic carbon; WT, wild-type maize LH1037; WL-73, LH1037 plant transformed with *BcWRKY1*; V3, the three lowest leaves have a visible collar; V9, nine leaves have collars present; R1, silking; R6, physiological maturity.

*∗* indicates a significant difference at *P* < 0.05 according to the LSD test (*n* = 3).

*∗∗* indicates a significant difference at *P* < 0.01 according to the LSD test (*n* = 3).

Note: only those traits with significant differences in a specific period are listed here.

**Table 4:** ANOVA of four enzyme activities in rhizosphere soils.

Enzyme activities	Source of variation	*P* value
Alkaline phosphatase	Year	0.87
	Soil variety	0.01^*∗∗*^
	Maize variety	0.90
	Stage × maize variety	0.00^*∗∗*^

Urease	Year	0.81
	Soil variety	0.00^*∗∗*^
	Maize variety	0.52
	Stage × maize variety	0.00^*∗∗*^

Dehydrogenase	Year	0.96
	Soil variety	0.87
	Maize variety	0.77
	Stage × maize variety	0.00^*∗∗*^

Sucrase	Year	0.56
	Soil variety	0.01^*∗∗*^
	Maize variety	0.88
	Stage × maize variety	0.00^*∗∗*^

Years: 2012, 2013, and 2014.

Soil varieties: saline soil and nonsaline soil.

Maize varieties: WT (wild-type maize LH1037) and WL-73 (LH1037 plant transformed with *BcWRKY1*).

Stages: V3 (the three lowest leaves have a visible collar), V9 (nine leaves have collars present), R1 (silking), and R6 (physiological maturity).

^*∗∗*^Significant source of variation (*P* < 0.01).

**Table 5:** ANOVA of seven physicochemical properties.

Physicochemical properties	Source of variation	*P* value
EC	Year	0.9972
	Soil variety	0.012^*∗*^
	Maize variety	0.156

pH value	Year	0.84
	Soil variety	0.001^*∗∗*^
	Maize variety	0.742

SAR value	Year	0.56
	Soil variety	0.001^*∗∗*^
	Maize variety	0.742

AN	Year	0.87
	Soil variety	0.052
	Maize variety	0.038^*∗*^

AP	Year	0.62
	Soil variety	0.002^*∗∗*^
	Maize variety	0.791

AK	Year	0.84
	Soil variety	0.033^*∗*^
	Maize variety	0.935

OC	Year	0.95
	Soil variety	0.012^*∗*^
	Maize variety	0.513

Years: 2012, 2013, and 2014.

Soil varieties: saline soil and nonsaline soil.

Maize varieties: WT (wild-type maize LH1037) and WL-73 (LH1037 plant transformed with *BcWRKY1*).

^*∗*^Significant source of variation (*P* < 0.05).

^*∗∗*^Significant source of variation (*P* < 0.01).
